# How We Know It Hurts: Item Analysis of Written Narratives Reveals Distinct Neural Responses to Others' Physical Pain and Emotional Suffering

**DOI:** 10.1371/journal.pone.0063085

**Published:** 2013-04-26

**Authors:** Emile Bruneau, Nicholas Dufour, Rebecca Saxe

**Affiliations:** Department of Brain and Cognitive Sciences, Massachusetts Institute of Technology, Cambridge, Massachusetts, United States of America; University of Tokyo, Japan

## Abstract

People are often called upon to witness, and to empathize with, the pain and suffering of others. In the current study, we directly compared neural responses to others' physical pain and emotional suffering by presenting participants (n = 41) with 96 verbal stories, each describing a protagonist's physical and/or emotional experience, ranging from neutral to extremely negative. A separate group of participants rated “how much physical pain”, and “how much emotional suffering” the protagonist experienced in each story, as well as how “vivid and movie-like” the story was. Although ratings of Pain, Suffering and Vividness were positively correlated with each other across stories, item-analyses revealed that each scale was correlated with activity in distinct brain regions. Even within regions of the “Shared Pain network” identified using a separate data set, responses to others' physical pain and emotional suffering were distinct. More broadly, item analyses with continuous predictors provided a high-powered method for identifying brain regions associated with specific aspects of complex stimuli – like verbal descriptions of physical and emotional events.

## Introduction

At the 1994 Olympics, the young running phenomenon Mary Decker turned the corner in the 3000-meter race she was favoured to win, and then suddenly collided with her running nemesis, Zola Budd, falling hard to the ground on her hip. A picture captures the moment as Mary Decker, tears streaming down her face and mouth open in anguish, watches as the runners continue without her. People who saw that event first hand, who have seen the Pulitzer prize-winning photograph, or even who just read this brief verbal description of the event, respond to two distinct (though related) aspects of Decker's experience: the physical pain in her injured body, and the emotional suffering as she watched her Olympic dreams recede in front of her.

In real life, misfortunes often combine physical pain and emotional suffering. Events that are emotionally painful without a direct physical cause (grieving over the loss of a loved one, or agonizing over unrequited love) are described in language borrowed from physical pain (“feeling like you were hit in the gut”, “love hurts”). Conversely, simple physical injuries nevertheless elicit strong emotions: fear, anger, anxiety, shame.

When watching or reading about these events, do we recognize another person's physical pain and understand their emotional suffering using a single unified neural system? Or are there distinct neural systems for these two processes? Recent neuroimaging studies have found evidence both hypotheses. On the one hand, a group of brain regions collectively called the ‘Shared Pain network,’ including parts of bilateral anterior insula (AI) and anterior middle cingulate cortex (AMCC), are recruited both when participants experience physical pain, and when they observe others experiencing similar pain [1]–[7] (but see [8], [9]). Activity in AI and AMCC is correlated with trial-by-trial measurements of the intensity of physical pain experienced [10] or observed [11]. The response in these regions is influenced by the affective aspects of painful experiences, and not just the sensory aspects (for more details see [12]). For example, activity in insula and AMCC is modulated by participants' anxiety and fear associated with anticipating pain, even prior to any actual painful sensation [13], [14]. Finally, there is evidence that these same regions are recruited when experiencing, or witnessing another person experience, purely “social” suffering, e.g. during exclusion from a social interaction [15], [16]. These results have been interpreted as evidence that AI and AMCC are the primary brain regions involved in responses to others' physical and emotional suffering.

On the other hand, other recent studies find that thinking about another person's feelings of guilt, embarrassment and/or grief does not elicit activity in AI or AMCC [17], [18]. Rather, thinking about another person's feelings seems to predominantly lead to activity in a region of medial prefrontal cortex (DMPFC). The DMPFC is active while participants read verbal stories describing individuals experiencing emotional loss [19], and while participants read stories or look at cartoons, and then make inferences about the characters' emotions [20], [21]. Individuals with more activity in DMPFC while observing others' suffering later offer more help to alleviate that suffering [16], [22], and individuals who reported more frequently helping friends in their daily lives (in a diary study) show greater DMPFC response to depictions of emotional suffering [23]. These results suggest that AI and AMCC are predominantly recruited when witnessing others in physical pain, and DMPFC is more implicated in empathic responses to emotional suffering.

One interpretive challenge for prior studies, however, may be that – as in our initial example – witnessing another person's misfortune often carries elements of *both* physical pain and emotional suffering. Watching someone receive an electric shock, or be poked by a painful needle, we may spontaneously anticipate their fear and sadness, as well as their physical discomfort. Hearing about someone heading into a terrifying final exam, we may spontaneously imagine the clenched gut and sweaty palms of their physical experience. How can these correlated features of experience be separated in an experiment?

The present study sought to disentangle the naturally confounded experiences of witnessing physical pain and emotional suffering using a parametric item-analysis [24]. In the study, participants read verbal stories that varied in how much physical pain and how much emotional suffering the protagonist endured. We obtained behavioral ratings of each story from a separate group of participants, on how much physical pain and emotional suffering the main character experienced, and how vivid the verbal description was; we then compared these ratings with brain responses to each story across participants.

These data represent a subset of the data that were analyzed previously [25] (see methods for details). As with most neuroimaging studies, stories in this previous analysis were assigned to categories based on the subjective judgment of the experimenter: for example, a story about a boy who falls while walking on a picket fence and is left hanging by his broken leg is categorized as “Physical Pain”, and a story about a father and son who find out that the child has terminal cancer is categorized as “Emotional Pain”. Within a condition, the intensity of pain and suffering inevitably varied, and this was treated as a source of noise: the story about the boy with the broken leg and a story about a girl who burns her finger on a hot light bulb are treated equally as “Physical Pain” items. However, this standard fMRI analysis technique ignores two important points: first, physical pain and emotional suffering are conflated in each category (the boy left hanging on the fence certainly felt physical pain, but also certainly suffered from the terror, embarrassment and confusion); and second, variability in the stories could help to illuminate brain regions that are sensitive to that variation, rather than merely being treated as noise. Therefore, in the present item analysis [26], [27] we use independent ratings to simultaneously characterize each item across multiple dimensions (e.g. both how much physical pain and how much emotional suffering each story depicts), and treat variability of items (e.g. burning a finger versus breaking a leg) within ‘conditions’ as an opportunity for additional statistical leverage on the effects of interest. Since so many phenomena of interest to social neuroscience are continuous rather than categorical (e.g. fear, disgust, pain), and inter-related rather than orthogonal (e.g. pain and suffering, shame and humiliation), this analysis strategy could be leveraged effectively in other studies involving complex social stimuli, and therefore could be of great utility to social cognitive neuroscience.

## Methods

### Participants

fMRI data were collected from 41 naïve right-handed participants (18–37 years old (mean 23.0±4.8 s.d.), 25 females). A separate group of fourteen participants (19–33 years old (mean 23.5±4.1 s.d.), 8 female) engaged in the localizer experiment. The study protocol was approved by MIT's Committee on the Use of Humans as Experimental Subjects, and was conducted at the Athinoula A. Martinos Imaging Center in the McGovern Institute for Brain Research at the Massachusetts Institute of Technology. All participants were proficient English speakers, had normal or corrected to normal vision, gave written informed consent, and were compensated for their time.

The item ratings were obtained from approximately 30 “workers” per story on Amazon's Mechanical Turk (mTurk), an online interface; stories were presented one at a time, and individual workers rated stories ad libitum. These workers gave informed consent to participate, and were compensated per story, in accordance with the requirements of MIT's Committee on the Use of Humans as Experimental Subjects.

### Design and Materials

Forty-eight verbal stories involving mild to extreme physical or emotional pain were constructed (“high pain/suffering”); a milder form of each story was also created, in which the characters and scene remained unchanged but the physical or emotional pain were eliminated or lessened (“low pain/suffering”). This resulted in 96 total stories. Stories varied in the depicted environment, the number of people involved, and age and gender of the protagonist, but were approximately matched for length (mean: 46.9 words ±3.5 s.d.) and complexity (Flesch reading level: 86.3±6.2 s.d.). (For sample stories, see Table 1; for full list of stimuli, see Methods S1).

**Table 1:** Sample stories.

Scenario	Pain	Suffering	Vivid
Kevin took his son Zack to the doctor for a checkup. The doctor did a series of tests and came back to talk to the father and son. The doctor told them that Zack has a rare form of cancer that they have no cure for. He gives Zack 6 months to live.	4.6	8.7	6.7
Bill was walking along a picket fence with his friend. Bill is in kindergarten and was trying to show his friend how fast he could walk. Bill stumbled and fell onto a sharp picket. The picket pierces his leg and Bill was left hanging on the fence.	8.2	6.3	5.8
Mark had wanted to ask Christy on a date for months. One day Mark walked up to her and asked her out. Christy said that she was not interested and walked off. Mark did not even have time to give her the flowers that he brought.	2.9	6.3	5.9
Liane was changing a lightbulb in her living room. Her roommate held a stool while Liane reached up to unscrew the old bulb. The light had been on all night, though, and it was very hot. When she grabbed the bulb it burned Liane's hand.	6.9	4.6	6.2
Lauren slept on a new pillow last night that was firmer than she was used to. Lauren has had back problems ever since she had a bicycle accident. Lauren woke up in the morning with no back pain and she did not have to take any Advil.	3.3	3.5	4.2

Representatives from 96 total stories used in the neuroimaging study. Each story was rated for “how much physical pain” the protagonist felt, “how much emotional suffering” the protagonist experienced, and “how vivid and ‘movie-like’” the story was.

An additional 48 stories involving false beliefs were also presented but were excluded from the analysis. In these stories, the protagonist's false beliefs always led to emotions (there were no stories in which a character experienced physical pain based on a false belief), and could therefore bias our analysis to find differences between emotional and physical stories, which truly reflected only the presence of false beliefs.

Half the participants in the study were told to “rate the pain/suffering” experienced by the protagonist of each story, and half the participants were told to “actively empathize” with the protagonist of each story [25]. We have previously reported that participants' explicit task had little effect on neural responses to these stimuli; we therefore average across both groups of participants in the current analysis.

#### Behavioral ratings

The 96 short stories were presented one at a time, using the online interface of mTurk. mTurk “workers” rated each story on three separate 9-point scales (anchored at “none at all” and “extreme”):

Pain: “How much physical pain was the main character in?”

Suffering: “How much emotional suffering did the main character experience?”

Vividness: “How vivid and ‘movie-like’ was this scenario to you?”

The 96 stories were each rated by 40 people for “pain” and “suffering”, and by 20 people for “vividness”. A worker's responses to an item were dropped if: 1. they gave any response except ‘9’ (“completely agree”) for a ‘catch’ question (“I read the story and answered all the questions honestly”), or 2. the response times were unreasonably fast (unrealistic reading rates of >600 words per minute). These restrictions resulted in the exclusion of approximately 15% of the responses. After excluding these data, each story was rated across the dimensions of “pain” and “suffering” by 26–40 people (mean 34.6±4.0 s.d.), and across the dimension of “vividness” by 12–20 people (17±2.1).

#### fMRI imaging

Participants in the fMRI study each read 48 of the 96 stories. The sample of items was approximately balanced across participants (to ensure every participant saw approximately 12 stories with the highest levels of emotional suffering and physical pain) but otherwise randomly chosen. The number of participants who saw each specific story was 19.9±3.8 s.d. (range: 11–29).

Each run consisted of 4 blocks, separated by 12 s fixation periods; within each block, 3 stories were presented for 16 s each, separated by 2 s. In total, each run contained 12 stories, 2 from each of the original six conditions, in counterbalanced order, and lasted 4.6 minutes; the experiment consisted of 6 total runs. While in the scanner, participants were asked to respond to each story using a MRI-safe 4-button box.

#### Image Acquisition and Analysis

Participants were scanned using a Siemens Magnetom Tim Trio 3T System (Siemens Solutions, Erlangen, Germany) in the Athinoula A. Martinos Imagining Center at the McGovern Institute for Brain Research at MIT using 30 4-mm-thick near axial slices with whole brain coverage (TR  = 2 s, TE  = 30 ms, flip angle  = 90). The experiment used a block design, and was modeled using a boxcar regressor.

MRI data were analyzed using SPM8 (http://www.fil.ion.ucl.ac.uk/spm/software/spm8/), and custom software. Each participant's data were motion corrected and normalized onto a common brain space (Montreal Neurological Institute, MNI, Template). Data were smoothed using a Gaussian filter (full width half maximum  = 5 mm).

For whole brain analyses, we first built a modified linear model of the experimental design to analyze the BOLD response in each voxel. The model included both covariates of interest (the stimulus items) and nuisance covariates – run effects, an intercept term, and global signal. We modeled each item as a box-car (matching the onset and duration of each story) convolved with a standard hemodynamic response function (HRF). Time-series data were subjected to a high-pass filter (128 Hz).

Average beta responses were calculated across participants for each story. Random Effects analysis was performed across the item-wise beta-images [26], using the ratings on each question (Pain, Suffering, Vividness) as user-defined regressors. These analyses used corrected p thresholds of p<0.05 in a voxel-wise analysis using Statistical non-Parametric Mapping (SnPM). Because these predictors were correlated, it was interesting to ask whether they nevertheless predict independent components of the neural response; therefore analyses were also conducted including all three regressors simultaneously in a single model, with p thresholds at k>10, p<0.001, uncorrected.

For regions of interest analyses, a set of ROIs was generated from a separate study in which participants directly viewed another person experiencing a painful and unpleasant electrical shock in real time. In this “Pain Localizer” study, participants in the scanner directly observed a confederate's hand as the confederate received a painful and unpleasant electric shock (versus a barely perceptible, non-painful shock) to the visible hand. Based on activity in this task, we identified 8 functional regions of interest (ROIs) involved in experiencing and/or perceiving pain or suffering: bilateral secondary sensory cortex (l and r SII), bilateral insula, anterior middle cingulate cortex (AMCC), bilateral thalamus, and dorsal medial prefrontal cortex (DMPFC) (for details, see Methods S1 and [25]). Then, the average response (i.e. beta value) within each ROI for the current participants was determined for each of the 96 stories.

Principal Component Analysis was applied to the 8×96 matrix of the average betas for the 96 stories in 8 ROIs. These components were then correlated with the behavioral ratings of Pain, Suffering and Vividness. Factor correlations were determined for each behavioral rating individually, and also for the residual of each rating after the other two ratings had been accounted for.

To ensure that the ratings of Pain and Suffering explained variance in the neural data beyond the variance that could be explained by the original binary categorization of the stories, we performed a two-step regression: first, we accounted for the variance in the item-wise neural data within the regions of interest using the categorical regressors of Pain (a ‘1’ for stories designed to focus on Physical Pain, and a ‘0’ for all other stories) and Suffering (a ‘1’ for stories designed to focus on Emotional Suffering, and a ‘0’ for all other stories). Second, we used the behavioral ratings of Pain and Suffering for each story as continuous regressors and determined if these regressors explained the residual variance.

## Results

### Behavioral results

Across stories, ratings of Pain, Suffering and Vividness were all significantly positively correlated with each other (Pain-Suffering: pearson's r = 0.43; Pain-Vivid: r = 0.52; Suffering-Vivid: r = 0.76, all p-values <0.001). We next tested whether these behaviorally inter-correlated features of the stories were represented in similar or distinct brain regions.

### Whole brain random effects analyses

**Figure 1:**
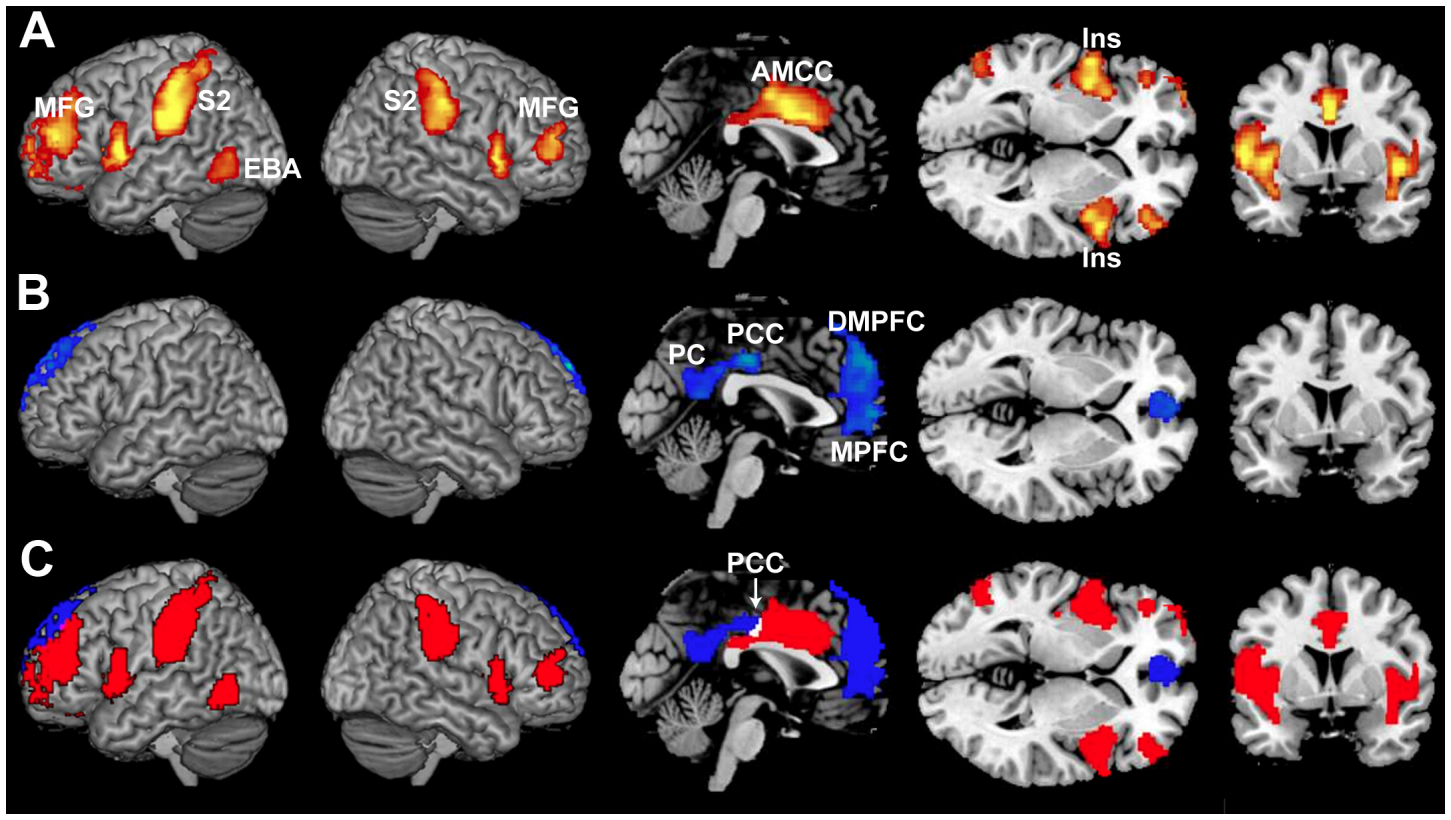
Item-wise correlations of brain activity with ratings of physical pain and emotional suffering. Regression analyses identified the brain regions where brain activity was most highly correlated with behavioral ratings of (A) “How much physical pain was the main character in?” (hot), and (B) “How much emotional suffering did the main character experience?” (cool). The brain regions where activity correlated with ratings of physical pain (A) include the bilateral insula cortex (Ins), anterior middle cingulate cortex (AMCC), bilateral middle frontal gyrus (MFG), bilateral secondary sensory regions (SII) and right extrastriate body area (EBA). The brain regions where activity correlated with ratings of emotional pain (suffering) (B) included the left dorsal striatum/anterior thalamus (Thal), precuneus (PC), posterior cingulate cortex (PCC), and regions in the medial prefrontal cortex (MPFC). Shown in (C) are both the regions where activity correlated with Pain (red), where activity correlated with Suffering (blue), and the conjunction of the two (white). All analyses are shown at p<0.05, corrected.

We performed three whole-brain item-wise Random Effects analyses, comparing neural responses across items to continuous behavioral ratings of Pain, Suffering and Vividness. Ratings of the character's physical Pain (Figure 1A) correlated positively with activity in the bilateral secondary sensory regions (SII), the anterior middle cingulate cortex (AMCC), bilateral insula cortex (Ins), middle frontal gyri (MFG) and a left lateral striate region, near the location of the extrastriate body area (EBA) [28]. Ratings of the character's emotional Suffering (Figure 1B) were positively correlated with activity in the posterior cingulate cortex (PCC), precuneus (PC), and dorsal, middle and ventral regions of the medial prefrontal cortex (MPFC) (Figure 1 and Table 2). At this threshold (p<0.05, corrected) story Vividness was not significantly correlated with activity in any brain region. At the more relaxed threshold of p<0.001, uncorrected, ratings of Vividness correlated with activity in small regions of the anterior thalamus/caudate bilaterally, the right middle insula, and the posterior cingulate cortex.

**Table 2:** Coordinates of peak brain activity for regressions of Pain, Suffering and Vividness.

Pain							
Cluster-level		Cluster-Voxel combo				Voxel-level	
k	*w*	*P_combo_*	x	y	z	peak *t*	Brain Area
3719	8.82	0.0006	−54	4	6	7.49	Left Insula
			−40	0	6	6.30	Left Insula
			−44	50	16	6.15	Left Middle Frontal Gyrus
1595	8.82	0.0006	52	10	−2	6.86	Right Insula
			44	4	−2	6.41	Right Insula
			56	10	6	5.82	Right Insula
2007	8.82	0.0006	0	6	30	6.79	Anterior Middle Cingulate Cortex
			2	14	30	6.38	Anterior Middle Cingulate Cortex
			0	2	38	6.12	Anterior Middle Cingulate Cortex
2870	8.82	0.0006	−62	−22	28	6.76	Left Secondary Sensory
			−56	−32	48	6.59	Left Secondary Sensory
			−62	−30	30	6.58	Left Secondary Sensory
1196	7.57	0.0022	64	−26	38	6.17	Right Secondary Sensory
			62	−30	50	5.63	Right Secondary Sensory
			68	−26	24	5.24	Right Secondary Sensory
249	6.68	0.0054	−28	36	−12	6.01	Left Orbitofrontal Gyrus
502	5.61	0.0158	48	50	12	5.54	Right Middle Frontal Gyrus
			50	44	4	5.08	Right Middle Frontal Gyrus
			42	56	24	4.76	Right Middle Frontal Gyrus
**Suffering**							
3148	8.42	0.0008	−8	50	22	6.09	MPFC
			6	58	36	5.75	DMPFC
			−4	36	62	5.00	Posterior DMPFC
1088	6.08	0.0102	−2	−20	40	5.32	Posterior Cingulate Cortex
			−10	−58	32	4.51	Left Precuneus
			−2	−52	18	4.25	Precuneus
**Vividness**							

MNI coordinates, t-value of the peak voxels in each cluster, and the brain regions that correspond to each peak for each of the contrasts used in the study. All analyses thresholded at p<0.05 (corrected).

No supra-threshold voxels at p<0.05, corrected.

To test whether any of these brain regions showed ‘shared’ activity for Pain and Suffering, we conducted a conjunction analysis (Figure 1C). Of the total number of voxels associated with either ratings of Pain and Suffering, only 0.2% (41/17027 voxels) were overlapping, all located in the posterior cingulate cortex (BA 23).

Because the ratings of Pain, Suffering, and Vividness were all positively correlated, some observed overlap between these regions may be explained by shared variance in the predictors. To examine this, we whole brain item-wise random effects analyses for each of the rating items separately, and a single whole brain item-wise random effects analysis at the same relaxed threshold (p<0.001, uncorrected) in which all three scales were included as simultaneous regressors. Relative to regressing each variable separately, the simultaneous model (Figure S1) showed a net *increase* in supra-threshold voxels for Pain (12523 voxels separate, 15970 voxels simultaneous) and Suffering (4595 voxels separate, 8397 voxels simultaneous) with no change in the number of clusters, but a net *decrease* in supra-threshold voxels for Vividness (695 voxels separate, 187 voxels simultaneous).

### Regions of Interest

We identified 8 ROIs based on responses to a Pain Localizer task in an independent group of participants: left and right secondary sensory regions (SII), left and right insula, left and right anterior thalamus, anterior middle cingulate cortex (AMCC) and dorsomedial prefrontal cortex (DMPFC). In each ROI, average beta values were extracted for each story.

In a principal component analysis (PCA) of the responses of these eight regions across items, the first principal component explained 60% of the variance. The DMPFC, left thalamus and right thalamus ROIs loaded positively on this component, while the remaining 5 ROIs (left and right SII, left and right insula, AMCC) loaded negatively (Table 3). Also, this first principle component was strongly positively correlated with ratings of Suffering (r = 0.35, p<0.0005), negatively correlated with ratings of Pain (r = −0.27, p<0.01), and uncorrelated with ratings of Vividness (r = 0.17, p = 0.11), even though ratings of Pain, Suffering, and Vividness were positively correlated across items. We also conducted this analysis using only the residual variance for each of these ratings that was unshared with the other two ratings. Again, the residual variance in Suffering and Pain were strongly correlated with the first principal component of ROI responses (Suffering: r = 0.38, p<0.0005; Pain: r = −0.44, p<0.0001), while Vividness was not (r = −0.01, p = 0.95).

**Table 3:** Principal component analysis (PCA) of brain responses to stories in regions of interest (ROIs) defined in an independent ‘pain empathy’ localizer.

Factor Loadings							
L SII	R SII	L Ins	R Ins	AMCC	LThal	RThal	DMPFC
−0.14	−0.16	−0.15	−0.13	−0.10	0.14	0.19	0.92

Shown are the factor loadings in the first factor of a PCA that used the average beta response to each of the 96 stories in each of the 8 ROIs. Together, this factor accounted for 60% of the variance. SII  =  secondary sensory cortex, Ins  =  insula, AMCC  =  anterior middle cingulate cortex, Thal  =  anterior thalamus, DMPFC  =  dorsomedial prefrontal cortex.

**Figure 2:**
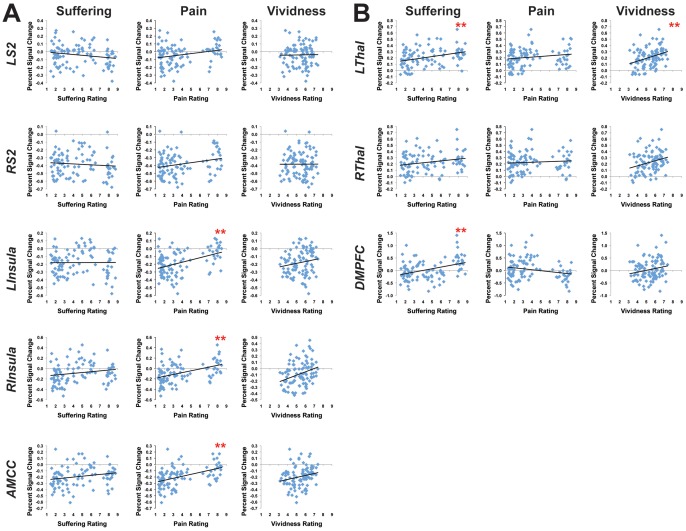
Ratings of Pain, Suffering and Vividness compared to brain activity in 8 ROIs. The average ratings of Physical Pain and Emotional Suffering experienced by the protagonist in each story, and the overall Vividness of the scene were compared to brain activity elicited for each story in 8 ROIs identified in a separate data set. Shown in (A) are the 5 ROIs that loaded negatively onto the first factor of the principal component analysis, and in (B) the 3 ROIs that loaded positively onto that first factor. Pain, Suffering and Vividness were rated on a scale from 1 (none) to 9 (extreme), and brain activity was measured as the average beta value within each ROI. ** p<0.002 (significant, correcting for multiple comparisons). SII  =  secondary sensory cortex, Ins  =  insula, AMCC  =  anterior middle cingulate cortex, Thal  =  anterior thalamus, DMPFC  =  dorsomedial prefrontal cortex.

To further explore the correlations between ratings of Pain, Suffering and Vividness and responses in individual ROIs, we conducted post-hoc pairwise correlation analyses. Due to the large number of regressions performed, we used a significance threshold that reflected a correction for multiple comparisons (p-value <0.002). Of the brain regions that loaded *negatively* on the first principal component (Figure 2A), ratings of Pain positively correlated with brain activity in left insula (pearson's r = 0.44, p<0.002), right insula (r = 0.43) and AMCC (r = 0.47), and showed a positive trend in lSII (r = 0.26, p<0.05) and rSII (r = 0.25, p<0.05); none of these brain regions were positively correlated with Suffering. Of the brain regions that loaded *positively* on the first principal component (Figure 2B), on the other hand, ratings of Suffering were significantly correlated with activity in the left anterior thalamus (r = 0.33) and DMPFC (r = 0.38), and showed a positive trend in the right anterior thalamus (r = 0.22, p<0.05); none of these brain regions were positively correlated with Pain, but the DMPFC showed a negative trend with ratings of Pain (r = −0.22, p<0.05).

Ratings of Vividness were positively correlated with activity in the left anterior thalamus (r = 0.33, p<0.002), and trended towards positive correlations with activity in the right insula (r = 0.30, p<0.05), right anterior thalamus (r = 0.27, p<0.05), and the AMCC (r  =  0.22, p<0.05) (Table 4).

**Table 4:** Item-wise correlations between brain activity and behavioral ratings within specific regions of interest.

	Suffering	Pain	Vivid
lSII	−0.19	0.26*	0.02
rSII	−0.09	0.25*	0.00
lIns	0.01	0.44**	0.17
rIns	0.19	0.43**	0.30*
aMCC	0.21*	0.47**	0.22*
lThal	0.33**	0.17	0.33**
rThal	0.21*	0.07	0.27*
dmPFC	0.38**	−0.22*	0.19

Behavioral ratings for each story for Pain, Suffering and Vividness were correlated with average brain activity in 8 regions of interest defined in an independent data set. SII  =  secondary sensory cortex, Ins  =  insula, AMCC  =  anterior middle cingulate cortex, Thal  =  anterior thalamus, DMPFC  =  dorsomedial prefrontal cortex.

* **p<0.05.**

** **p<0.002.**

After accounting for variance explained by the original binary categories of the stimuli (physical pain, emotional suffering), we found that the continuous measures still explained significant variance in the left and right SII (p-values <0.01), the right insula (p<0.01), the aMCC (p<0.001), the dmPFC (p<0.05), and provided marginal additional predictive power for the left thalamus and left insula (p-values <0.10). The continuous regressors failed to explain residual variance only in the right thalamus (p = 0.4).

## Discussion

In our stimuli, as in many real life experiences, the physical pain, emotional suffering, and vividness of the experience were all positively correlated. Positive correlations between distinct features of complex social stories raise a challenge for neuroimaging experiments. In the current experiment, we addressed that challenge using parametric item analysis. This analysis allowed us to go beyond the limits of both standard subtraction techniques, which require that stimuli be assigned to a small number of discrete conditions, and of regions of interest analyses, which focus on constrained brain regions, to look at the association and dissociation between continuous cognitive processes.

Using item analysis, we found that different brain regions are correlated with the amount of physical pain versus emotional suffering depicted in verbal stories. Across 96 stories, the amount of physical pain experienced by the protagonist predicted neural activity in bilateral secondary sensory regions, bilateral insulae, anterior middle cingulate cortex, bilateral middle frontal gyri and a left lateral extrastriate region, possibly the left EBA [29]. By contrast, the amount of emotional suffering depicted in the same stories was correlated with activity in the precuneus, posterior cingulate, and medial prefrontal cortex. Converging results appeared in whole brain and regions of interest analyses, and in spite of the fact that ratings of Pain, Suffering and Vividness were positively correlated in the stimuli. These results therefore strongly suggest that people have distinct neural responses to other people experiencing physical pain versus emotional suffering.

The parametric item analysis has multiple methodological advantages over more standard subject-wise analyses used in our prior papers [19], [25]: (1) the behavioral ratings used to predict neural response to each item were assigned by naive participants (rather than by the experimenter), (2) multiple correlated dimensions of the stimuli could be studied simultaneously, (3) we explicitly tested whether the observed relationships can be generalized beyond the current sample of stimuli, by treating items as a random effect [24], [26], [27] (4) the statistical power of the analyses is related to the number of items (n = 96), which is greater than the number of participants (n = 41), and (5) variance within a ‘condition’ could be used as statistical leverage, rather than ignored as noise. Item analysis can thus provide strong evidence for the association between a brain region's activity and intrinsic dimensions of high-level complex stimuli, like vignettes about other people (rather than participants' specific experiences of the stimuli).

Consistent with our previous analyses of these data [25], the current results show that simply reading about another person's physical pain can produce activity in the ‘Shared Pain network’. In prior research, these regions (especially bilateral insula and middle cingulate cortex) were recruited when participants viewed images of body parts threatened by needles and knives [4], [7], [30], [31], watched videos of people's faces while they undergo painful physical therapy [5], [11], were cued that a loved one was receiving an electric shock [6], or were reminded of a documentary about another's pain [3], [32]. In the current experiment, these same regions' responses were robustly correlated with continuous ratings of physical pain experienced by a (fictional) stranger in a verbal story. It is particularly interesting that we observed significant responses in right and left secondary sensory cortex to verbal stories (although note that the responses in the sensory regions were low overall); just imagining sensory experiences appears to be sufficient to modulate sensory cortices [33]. These results may have practical implications, since written stories can be transmitted so much further, and faster, than direct dyadic social interactions.

One striking feature of these results is that they suggest a functional divide between two subsets of the ‘Shared Pain network’. We identified 8 regions of interest based on a standard Pain Localizer task, in which participants experienced, and directly witnessed another person experiencing, painful and unpleasant electric shocks to the hand. This localizer is most likely to identify brain regions associated with the perception of acute, temporary, concrete, discrete, physical pain. For this reason it is particularly interesting that all of these regions are modulated by verbal stories describing painful experiences that are longer lasting, more distant in space and time and presented more abstractly – and for some regions, experiences that are more emotionally than physically painful.

Interestingly, rather than being explained by 2 separate components of equal weight, the data in the ROIs were best described by a single component of the regions' responses across items, which accounted for 60% of the variance. This component had a positive loading of left thalamus, right thalamus and DMPF, and a negative loading of bilateral insula, bilateral sensory regions, and the AMCC. This component in turn correlated positively with ratings of emotional Suffering and negatively with ratings of physical Pain, but was not correlated with ratings of story Vividness. While the overall PCA suggests that activity in the ROIs is best explained by a single, anti-correlated component, follow-up pairwise correlations within each ROI indicated that most regions were correlated with either the physical Pain or the emotional Suffering ratings. Analysis within the ROIs showed that only the middle cingulate region showed any hint of a positive correlation with both dimensions, and only the DMPFC showed a significant positive correlation with one (Suffering) and a *negative* correlation with the other (Pain).

The distinction between the two sets of brain regions responding to others physical pain versus emotional suffering may reflect distinct evolutionary histories. It is possible that responses to others' physical pain evolved earlier, followed by a second system that evolved in evolutionarily more recent regions (i.e. prefrontal cortex) as human social cognition developed. If, as suggested by the PCA, these two neural responses are anti-correlated, rather than simply uncorrelated, this could also be adaptive. It may be prudent to prioritize attention to another's physical pain over their emotional suffering until an immediate physical threat is addressed: coming upon the scene of a child hanging by their leg on a picket fence, or finding a relative gored by a wild animal, first attending to liberating them from the physically dangerous situation, and then addressing their emotional trauma may be more likely to save their lives. Further studies, potentially employing temporally sensitive methods like EEG or MEG, may help to resolve this issue of uncorrelated versus anti-correlated activity in the brain regions responsive to others' physical Pain and emotional Suffering.

How do the current results fit with the previous literature? In apparent contrast to the dissociation observed here, multiple prior studies have reported simultaneous activity in regions that we found to be uncorrelated with each other in the current analyses, such as AI, AMCC and DMPFC [1], [3], [11], [34]. We suggest that in those stimuli, as in real life, physical pain almost always includes some aspect of emotional suffering, so both processes may have been initiated in these previous studies. In our story stimuli, ratings of pain and suffering were strongly positively correlated. In the story empathy experiment we were able to distinguish these regions, by using a continuous parametric item analysis to separate correlations with emotional suffering versus physical pain in the same stimuli.

Our results converge with considerable prior evidence that regions in the MPFC are critical for affective perspective-taking [35]–[37]. DMPFC activity is observed when stimuli elicit a strong sense of a target's emotions – including people's faces [1], [5], [11] or the context of their suffering [5]. By contrast, just seeing images of a body part threatened by a needle, for example, does not lead to activity in MPFC [4], [7], [30], [31]. Similarly, a recent meta-analysis found that regions in the MPFC were recruited when a physically present live person was subjected to a painful experience (as it was in our Pain Localizer experiment), but not when similar injuries were depicted in photographs (note that the MPFC region in the current analysis is closer to the region labeled “VMPFC” than “DMPFC”, in the meta-analysis [32]).

Identifying the MPFC as a region that recognizes emotional suffering, but not physical pain, provides an interesting interpretation of a prior result on inter-group empathic failures. Black and white Americans, looking at images of other black and white Americans after a natural disaster (Hurricane Katrina), showed activity in both ‘pain’ (e.g. insula) and ‘suffering’ (e.g. MPFC) brain regions, but MPFC activity was reduced for outgroup targets [22]. In another study, neural responses in Arabs, Israelis and South Americans to stories involving ingroup, conflict outgroup and ‘distant’ outgroup protagonists experiencing either pain or suffering was examined. Activity in the DMPFC and VMPFC was reduced when reading about a distant outgroup member's emotional suffering [19]. These studies suggest that neural responses to observed emotional suffering may be more affected by inter-group differences than responses to physical pain.

The one area where brain activity correlated with both Pain and Suffering measures was a small region in the posterior cingulate cortex (PCC; Brodmann's Area 23). Cytoarchitectural data and functional connectivity suggest that the cingulate is a highly compartmentalized structure [38], [39]: while the AMCC (BA 24) has numerous thalamic inputs and is strongly associated with first and second-hand pain, the PCC (BA 23) immediately caudal to the AMCC shares reciprocal connections with high-level visual cortex [38], and this particular region of the PCC has been shown to activate when making moral judgments, particularly if those judgments are difficult [40], [41]. In the present study, PCC activity while making judgments about others' pain/suffering was sensitive to the extremity of another's suffering. Together, this suggests that the PCC is sensitive to rating others' emotional experiences per se, or to enhanced imagination of these emotional experiences as the severity increases. A more detailed analysis would be necessary to determine if the slight overlap of Pain and Suffering in this region represents a meaningful recruitment across conditions or a recruitment of different neural populations within the same region.

## Conclusion

In sum, using both whole brain and region of interest item-analyses, we found that the brain regions associated with the ‘Shared Pain network’ (secondary sensory, anterior middle cingulate and bilateral insulae) were recruited more for stories involving physical pain, while a distinct set of brain regions (especially in medial prefrontal cortex) were recruited more for stories involving emotional suffering. These brain regions thus seem to be involved, respectively, in representing another person's physical pain and emotional suffering. These neural systems may therefore provide a foundation for two distinct aspects of human empathy. Note, though, that the neural response measured here represents just the first steps in a full-blown empathic response [42]. While a neural representation of another's pain and suffering may precipitate empathic concern and helping behavior, this pro-social response is by no means inevitable. Understanding if someone is suffering is presumably just as important to an interrogator as it is to a social worker: representing another's pain and suffering could also be the first step to exploitation or even feeling delight. Determining which part of the activity observed here, or which additional downstream responses, represent true empathic concern will be a focus of future research.

## Supporting Information

Figure S1 **Whole brain item-analysis using ratings of Pain, Suffering and Vividness as simultaneous regressors.** (**A**) Ratings of Pain (hot), (**B**) ratings of Suffering (cool), and (**C**) ratings of Vividness (green).
(JPG)

Methods S1 (DOCX)

## References

[1] Botvinick M, Jha A, Bylsma L, Fabian S, Solomon P, et al (2005) Viewing facial expressions of pain engages cortical areas involved in the direct experience of pain. Neuroimage 25: 312–319. 15734365 10.1016/j.neuroimage.2004.11.043

[2] Gu X, Han S (2007) Attention and reality constraints on the neural processes of empathy for pain. Neuroimage 36: 256–267. 17400480 10.1016/j.neuroimage.2007.02.025

[3] Immordino-Yang M, McColl A, Damasio H, Damasio A (2009) Neural correlates of admiration and compassion. Proceedings of the National Academy of Sciences 106: 8021. 10.1073/pnas.0810363106 PMC2670880 19414310

[4] Jackson P, Meltzoff A, Decety J (2005) How do we perceive the pain of others? A window into the neural processes involved in empathy. Neuroimage 24: 771–779. 15652312 10.1016/j.neuroimage.2004.09.006

[5] Lamm C, Batson C, Decety J (2007) The neural substrate of human empathy: effects of perspective-taking and cognitive appraisal. Journal of cognitive neuroscience 19: 42–58. 17214562 10.1162/jocn.2007.19.1.42

[6] Singer T, Seymour B, O'Doherty J, Kaube H, Dolan R, et al (2004) Empathy for pain involves the affective but not sensory components of pain. Science 303: 1157–1162. 14976305 10.1126/science.1093535

[7] Xu X, Zuo X, Wang X, Han S (2009) Do you feel my pain? Racial group membership modulates empathic neural responses. Journal of Neuroscience 29: 8525–8529. 19571143 10.1523/JNEUROSCI.2418-09.2009 PMC6665679

[8] Corradi-Dell'Acqua C, Hofstetter C, Vuilleumier P (2011) Felt and seen pain evoke the same local patterns of cortical activity in insular and cingulate cortex. The Journal of Neuroscience 31: 17996–18006. 22159113 10.1523/JNEUROSCI.2686-11.2011 PMC6634156

[9] Morrison I, Downing P (2007) Organization of felt and seen pain responses in anterior cingulate cortex. Neuroimage 37: 642–651. 17588777 10.1016/j.neuroimage.2007.03.079

[10] Peyron R, Laurent B, Garcia-Larrea L (2000) Functional imaging of brain responses to pain. A review and meta-analysis (2000). Neurophysiologie Clinique/Clinical Neurophysiology 30: 263–288. 11126640 10.1016/s0987-7053(00)00227-6

[11] Saarela M, Hlushchuk Y, Williams A, Schurmann M, Kalso E, et al (2007) The compassionate brain: humans detect intensity of pain from another's face. Cerebral Cortex 17: 230–237. 16495434 10.1093/cercor/bhj141

[12] Fitzgibbon BM, Giummarra MJ, Georgiou-Karistianis N, Enticott PG, Bradshaw JL (2010) Shared pain: from empathy to synaesthesia. Neuroscience & Biobehavioral Reviews 34: 500–512. 19857517 10.1016/j.neubiorev.2009.10.007

[13] Atlas LY, Bolger N, Lindquist MA, Wager TD (2010) Brain mediators of predictive cue effects on perceived pain. The Journal of Neuroscience 30: 12964. 20881115 10.1523/JNEUROSCI.0057-10.2010 PMC2966558

[14] Wiech K, Lin C, Brodersen KH, Bingel U, Ploner M, et al (2010) Anterior insula integrates information about salience into perceptual decisions about pain. The Journal of Neuroscience 30: 16324. 21123578 10.1523/JNEUROSCI.2087-10.2010 PMC6634837

[15] Eisenberger N, Lieberman M, Williams K (2003) Does rejection hurt? An fMRI study of social exclusion. Science 302: 290. 14551436 10.1126/science.1089134

[16] Masten CL, Morelli SA, Eisenberger NI (2010) An fMRI investigation of empathy for social pain and subsequent prosocial behavior. Neuroimage. 10.1016/j.neuroimage.2010.11.060 21122817

[17] Takahashi H, Matsuura M, Yahata N, Koeda M, Suhara T, et al (2006) Men and women show distinct brain activations during imagery of sexual and emotional infidelity. Neuroimage 32: 1299–1307. 16829139 10.1016/j.neuroimage.2006.05.049

[18] Takahashi H, Yahata N, Koeda M, Matsuda T, Asai K, et al (2004) Brain activation associated with evaluative processes of guilt and embarrassment: An fMRI study. Neuroimage 23: 967–974. 15528097 10.1016/j.neuroimage.2004.07.054

[19] Bruneau EG, Dufour N, Saxe R (2012) Social cognition in members of conflict groups: behavioural and neural responses in Arabs, Israelis and South Americans to each other's misfortunes. Phil Trans R Soc B 367: 717–730. 22271787 10.1098/rstb.2011.0293 PMC3260847

[20] Hooker C, Verosky S, Germine L, Knight R, D'Esposito M (2010) Neural activity during social signal perception correlates with self-reported empathy. Brain Research 1308: 100–113. 19836364 10.1016/j.brainres.2009.10.006 PMC2798147

[21] Hooker CI, Verosky SC, Germine LT, Knight RT, DíEsposito M (2008) Mentalizing about emotion and its relationship to empathy. Social cognitive and affective neuroscience 3: 204. 19015112 10.1093/scan/nsn019 PMC2566770

[22] Mathur V, Harada T, Lipke T, Chiao J (2010) Neural basis of extraordinary empathy and altruistic motivation. Neuroimage 51: 1468–1475. 20302945 10.1016/j.neuroimage.2010.03.025

[23] Rameson LT, Morelli SA, Lieberman MD (2011) The Neural Correlates of Empathy: Experience, Automaticity, and Prosocial Behavior. Journal of cognitive neuroscience: 1–11. 10.1162/jocn_a_00130 21878057

[24] Clark HH (1973) The language-as-fixed-effect fallacy: A critique of language statistics in psychological research. Journal of verbal learning and verbal behavior 12: 335–359.

[25] Bruneau EG, Pluta A, Saxe R (2012) Distinct roles of the ‘Shared Pain’and ‘Theory of Mind’networks in processing others' emotional suffering. Neuropsychologia 50: 219–231. 22154962 10.1016/j.neuropsychologia.2011.11.008

[26] Bedny M, Aguirre GK, Thompson-Schill SL (2007) Item analysis in functional magnetic resonance imaging. Neuroimage 35: 1093–1102. 17346988 10.1016/j.neuroimage.2007.01.039

[27] Dodell-Feder D, Koster-Hale J, Bedny M, Saxe R (2011) fMRI item analysis in a theory of mind task. Neuroimage 55: 705–712. 21182967 10.1016/j.neuroimage.2010.12.040

[28] Downing PE, Jiang Y, Shuman M, Kanwisher N (2001) A cortical area selective for visual processing of the human body. Science 293: 2470–2473. 11577239 10.1126/science.1063414

[29] Downing PE, Jiang Y, Shuman M, Kanwisher N (2001) A cortical area selective for visual processing of the human body. Science 293: 2470. 11577239 10.1126/science.1063414

[30] Han S, Fan Y, Xu X, Qin J, Wu B, et al (2009) Empathic neural responses to others' pain are modulated by emotional contexts. Human brain mapping 30: 3227–3237. 19235883 10.1002/hbm.20742 PMC6870998

[31] Morrison I, Lloyd D, Di Pellegrino G, Roberts N (2004) Vicarious responses to pain in anterior cingulate cortex: Is empathy a multisensory issue? Cognitive, Affective, & Behavioral Neuroscience 4: 270. 10.3758/cabn.4.2.270 15460933

[32] Lamm C, Decety J, Singer T (2010) Meta-analytic evidence for common and distinct neural networks associated with directly experienced pain and empathy for pain. Neuroimage 54: 2492–2502. 20946964 10.1016/j.neuroimage.2010.10.014

[33] Keysers C, Wicker B, Gazzola V, Anton J, Fogassi L, et al (2004) A Touching Sight: SII/PV Activation during the Observation and Experience of Touch. Neuron 42: 335–346. 15091347 10.1016/s0896-6273(04)00156-4

[34] Zaki J, Ochsner K, Hanelin J, Wager T, Mackey S (2007) Different circuits for different pain: Patterns of functional connectivity reveal distinct networks for processing pain in self and others. Social neuroscience 2: 276–291. 18633819 10.1080/17470910701401973 PMC2913618

[35] Shamay-Tsoory S, Aharon-Peretz J, Perry D (2008) Two systems for empathy: a double dissociation between emotional and cognitive empathy in inferior frontal gyrus versus ventromedial prefrontal lesions. Brain. 10.1093/brain/awn279 18971202

[36] Shamay-Tsoory S, Tibi-Elhanany Y, Aharon-Peretz J (2006) The ventromedial prefrontal cortex is involved in understanding affective but not cognitive theory of mind stories. Social neuroscience 1: 149–166. 18633784 10.1080/17470910600985589

[37] Schnell K, Bluschke S, Konradt B, Walter H (2010) Functional relations of empathy and mentalizing: An fMRI study on the neural basis of cognitive empathy. Neuroimage. 10.1016/j.neuroimage.2010.08.024 20728556

[38] Vogt BA (2005) Pain and emotion interactions in subregions of the cingulate gyrus. Nature Reviews Neuroscience 6: 533–544. 15995724 10.1038/nrn1704 PMC2659949

[39] Yu C, Zhou Y, Liu Y, Jiang T, Dong H, et al.. (2010) Functional segregation of the human cingulate cortex is confirmed by functional connectivity based neuroanatomical parcellation. Neuroimage. 10.1016/j.neuroimage.2010.11.018 21073967

[40] Greene JD, Nystrom LE, Engell AD, Darley JM, Cohen JD (2004) The neural bases of cognitive conflict and control in moral judgment. Neuron 44: 389–400. 15473975 10.1016/j.neuron.2004.09.027

[41] Raine A, Yang Y (2006) Neural foundations to moral reasoning and antisocial behavior. Social cognitive and affective neuroscience 1: 203. 18985107 10.1093/scan/nsl033 PMC2555414

[42] Batson C (2009) These things called empathy: eight related by distinct phenomena. In: Decety J, Ickes W, editors. The social neuroscience of empathy. Cambridge, MA: MIT Press. 3–15.

