# Supplementary figures and images for: How We Know It Hurts: Item Analysis of Written Narratives Reveals Distinct Neural Responses to Others' Physical Pain and Emotional Suffering
Source: PLoS One. 2013 Apr 26;8(4):e63085. doi: 10.1371/journal.pone.0063085 (PMC3637309; doi:10.1371/journal.pone.0063085)

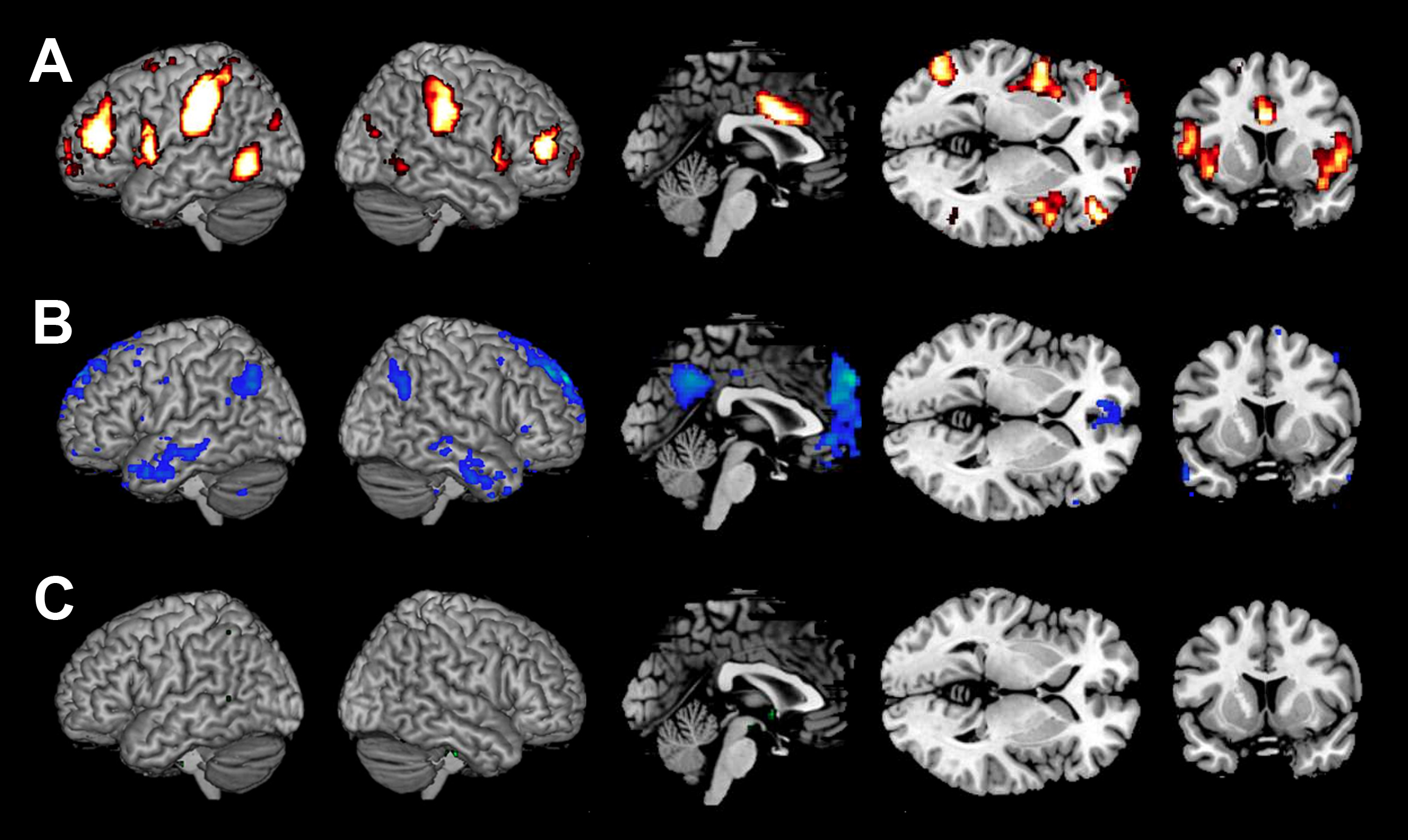

Supplement: Figure S1 — Whole brain item-analysis using ratings of Pain, Suffering and Vividness as simultaneous regressors. (A) Ratings of Pain (hot), (B) ratings of Suffering (cool), and (C) ratings of Vividness (green). (JPG) [file pone.0063085.s001.jpg]
