# Supplementary material for: How We Know It Hurts: Item Analysis of Written Narratives Reveals Distinct Neural Responses to Others' Physical Pain and Emotional Suffering
Source: PLoS One. 2013 Apr 26;8(4):e63085. doi: 10.1371/journal.pone.0063085 (PMC3637309; doi:10.1371/journal.pone.0063085)
Supplement: Methods S1 — (DOCX) [file pone.0063085.s002.docx]

**Methods:**

*Full List of Stimuli*

**Low to High Pain/Suffering scenarios**

1. Joe was playing soccer with his friends. He slid in to steal the ball away, but his cleat stuck in the grass and he rolled over his ankle, breaking his ankle and tearing the ligaments. His face was flushed as he rolled over.
2. Ethan was building a small shed. He picked up the hammer to drive a nail into a board, but missed and hit his thumb, crushing his thumb and ripping off half the thumbnail. Ethan held his thumb as the blood seeped through.
3. Roger was walking to school when he heard a friend behind him call his name. Roger turned to respond, but just then tripped and stumbled over some wood on the ground. Roger fell forward and impaled his hand on a rusty nail in the wood.
4. Liane was changing a lightbulb in her living room. Her roommate held a stool while Liane reached up to unscrew the old bulb. The light had been on all night, though, and it was very hot. When she grabbed the bulb it burned Liane’s hand.
5. Chris was chopping vegetables for dinner. When his friend asked him a question, Chris turned to respond and slipped with the knife. The knife cut a huge slice in his finger that went to the bone.
6. Leslie was running through her house playing tag with her friend. After going through a doorway, Leslie slammed the door behind her, but her finger was caught in the door. When they opened the door two of her fingers were broken.
7. Annie was in a park with her mother when a bee landed on her hand. Annie could feel the tickle of the bee legs walking on her. Then the bee stung Annie, injecting its poison into her hand. Annie shook her hand and ran to her mom.
8. Roy was at the beach with his family. After a day of playing catch and running in the sand, Roy started walking back to the car. Buried in the sand was a piece of broken glass. Roy stepped on the glass and it sliced the bottom of his foot.
9. Cindy was making dinner for her family. She pulled the pot of spaghetti off the stove and walked over to the sink to drain it. The pot slipped in her grasp and spilled boiling water on her arm and leg. Cindy rushed to the freezer to get some ice.
10. Chad was collecting wood to build a tree house. Chad picked up a long plank of old wood that was perfect for the floor. The wood slid in his hand and a splinter came off. A big splinter slid right underneath his fingernail and broke off.
11. Julia was sitting on a folding chair in her new room working at her desk. Julia needed to shift closer to her desk so she reached down and pulled the chair forward. Just then the chair collapsed and Julia’s finger was caught in the folding metal legs.
12. Lauren slept on a new pillow last night that was firmer than she was used to. Lauren has had back problems ever since she had a bicycle accident. Lauren woke up in the morning with a massive headache and she was out of Advil.
13. Jay has a pet rabbit that he keeps in a cage. One day he fed the rabbit some carrots and then put the rabbit away. As he was walking past the cage his bare leg caught on a wire that was sticking out. It left a long deep scratch along his thigh.
14. Bill was walking along a picket fence with his friend. Bill is in kindergarten and was trying to show his friend how fast he could walk. Bill stumbled and fell onto a sharp picket. The picket pierces his leg and Bill was left hanging on the fence.
15. Cathy was on an Island in the South Pacific for her honeymoon. She walked down the beach and into the warm water. As Cathy waded out she stepped on a stone fish which stuck its poisonous stinger into her foot. Cathy yelled and pulled her foot up.
16. Steve and his friend were riding bicycles together. Half way down a hill Steve realized that his brakes were broken. At the bottom of the hill Steve went off the road and hit a tree with his shoulder. He sat on the ground cradling his broken arm.
17. Suzie was riding in a cab to meet some friends for dinner. When she arrived at the restaurant Suzie opened the door and began to step out. Just then a child walking by accidentally bumped the door and it closed on Suzie, smashing her leg.
18. Eric was hiking in the mountains with his girlfriend. At a stream crossing Eric watched his girlfriend hop across the rocks and then began to follow. He slipped on the first rock and his shin came down hard on it, ripping off a swath of skin.
19. Angela was putting up posters in her new room. It is early Saturday morning and she was in her pajamas. As Angela stood back, she stepped on a thumb tack that had fallen on the ground. The thumb tack went into her heel and hit the bone.
20. Sonya was playing tag at school with her friends. While running around a corner, Sonya slipped on some gravel and fell. Her knee was scraped and had gravel imbedded in it. Sonya grabbed her bloody knee with both hands and started crying.
21. Larry was going to his first day of a new job. The job starts very early so Larry was extremely tired. Larry made himself some coffee and took a big drink. The coffee was still boiling hot and Larry felt the soft tissue inside his mouth burn.
22. Brian was at home when a friend called to him from outside. Brian raised the window to talk to his friend. Suddenly the window came down and pinned Brian’s fingers underneath the frame. Brian had to free his fingers by pulling them out.
23. Lois was skiing for the first time. She was learning how to go down the easy slopes and was trying to keep her legs together. On her first run down a harder slope her legs came apart and one knee twisted around. Lois cried out.
24. Oscar was doing the dishes after dinner. He was talking with his friends while his hands were in the soapy water. Then Oscars hand hit a sharp knife. The knife cut deep into the skin between his fingers and the cut burned in the dirty water.
25. Ron and his wife recently became a foster parents to a 10 year old girl. Ron promises her that he will be at a concert that she is playing in. On the way to the concert Ron is caught in traffic. When he arrives the concert is already over.
26. Aaron was going to visit his father. There was so much that he had never told his father, and he finally wanted to let him know how much he cared. When he got to his parents house an ambulance was there. His father had died in his sleep.
27. Diane has a son with Autism. Her son is often very uncomfortable with human contact and prefers to play on his computer. Since her husband left, all Diane has is her son. Diane went to her son to hug him and he screamed and pushed her away.
28. Shelley recently applied to Oxford; she has dreamed of going there all her life. One day a letter came from Oxford and Shelley tore it open. The letter was a rejection. Shelley ran into her room and began sobbing.
29. Mark is an autistic child who was just given his first dog. He loves the dog very much and considers it his best friend. One day Mark’s father wakes him up and tells him that the dog ran away. Mark sits on his bed rocking back and forth.
30. John was on a hike with his girlfriend. He had an engagement ring in his pocket and at a beautiful overlook he proposed marriage. His girlfriend said that she could not marry him and began crying. John sat on a rock and looked at the ring.
31. Monica was visiting Anna, who had been her best friend for 60 years. When Monica arrived at her house, Anna’s son said that Anna had had a massive stroke and they were not sure if she would survive. Monica sat down on the front porch.
32. Mark had wanted to ask Christy on a date for months. One day Mark walked up to her and asked her out. Christy said that she was not interested and walked off. Mark did not even have time to give her the flowers that he brought.
33. Rose was planning her upcoming wedding. She received a call from her fiancee. He told her that he was leaving town and could not go through with the wedding. Rose hung up the phone and dropped the wedding catalogue she was reading.
34. Kevin took his son Zack to the doctor for a checkup. The doctor did a series of tests and came back to talk to the father and son. The doctor told them that Zack has a rare form of cancer that they have no cure for. He gives Zack 6 months to live.
35. Katie was giving a piano recital and was waiting her turn. All the children performing walked off the stage to their parents when they finish. Katies parents died the previous year. When she finished her recital Katie sat looking at her hands.
36. Lara just moved and is trying to make friends at a new school. While Lara was sitting in one of the bathroom stalls, a number of girls come in. The girls began to talk about how much they disliked Lara and then they leave. Lara sat silently.
37. Lissa was excited to have a solo in her upcoming performance. Her father promised her that he will be there in the front row for the concert. When the concert comes Her father does not show up. After the show Lissa sat alone on the deserted stage.
38. Chad plays on a soccer team. After one of their big games Chad asks his teammates if they want to go get some ice cream. All of his friends tell him that they are going out with their fathers. Chad’s father forgot about the game and did not show up.
39. Karen was pregnant and going in for her first check-up. The doctor ran a number of tests and came back in to talk with her. The doctor told Karen that her baby had a rare disease and had died. Karen pleaded with the doctor to help her.
40. Lucas is a painter who just moved all of his beloved paintings to a new studio. One day on his way to the studio he hears fire engines. Lucas drives up to find that his studio is on fire; all of his work has been destroyed.
41. Mike is very much in love with his girlfriend. One day he goes to surprise her at work for lunch. Through the window of the building he sees his girlfriend walking with another man. She stops and gives the man a long kiss.
42. Alison lives by herself. Every week her son comes to visit her and they go out to lunch. Her son just lost his job and is arguing with his wife. One day Alison gets a call from her son who tells her that he has been diagnosed with cancer.
43. Nina lives with her parents in a small house. Lately her parents have been fighting a lot. They try to keep it away from Nina, but she can hear it. One day Nina’s father tells her that he and her mother are getting divorced and he is moving out.
44. Rudy had worked hard to get the job he has now. He can finally take care of his son and has almost saved enough to give him the gifts he wants for his birthday. Today he was called in to the office and his boss told him that he was fired.
45. Judy lives with her teenage daughter. Her daughter wants to have new friends and invites a number of people to her 15th birthday party. Nobody shows up to the birthday. Judy’s daughter goes into her room to cry and Judy stands helplessly.
46. Brett was taking his children to the family cabin. Brett was going to show his children all the family pictures and history. When they arrived the cabin was surrounded by fire trucks. Last night the cabin had burned to the ground.
47. Cynthia lives with her husband and step-daughter, Julia. She loves Julia very much and often tries to help with her homework. One day Julia jumped up from her work and shouted to Cynthia that she hates her. Cynthia sat alone at the table.
48. Bobby is new to school and joined the volleyball team. Tonight was their first match. At the end of a close game the ball came right to Bobby for an easy play. He missed the ball and they lost the game. His teammates groaned and walked off the court.

**No to Low Pain/Suffering scenarios**

1. Joe was playing soccer with his friends. He slid in to steal the ball away, and he kicked the ball away from the opposing player, got to his feat and began dribbling down the field. His face was flushed as he ran.
2. Ethan was building a small shed. He picked up the hammer to drive a nail into a board, drove it all the way through in a few hits, making the head of the nail flush with the wall. Ethan held the next board and picked up another nail.
3. Roger was walking to school when he heard a friend behind him call his name. Roger turned to respond, and then stepped over some wood in front of him. Roger stopped and picked up a long, rusty nail from the wood.
4. Liane was changing a lightbulb in her living room. Her roommate held a stool while Liane reached up to unscrew the old bulb. The new bulb was already broken, so Liane picked up a third bulb and screwed it in instead.
5. Chris was chopping vegetables for dinner. When is friend asked him a question, Chris turned to respond and put the knife in the sink. The knife clattered into the bowls and sank to the bottom of the water.
6. Leslie was running through her house playing tag with her friend. After going through a doorway, Leslie slammed the door behind her, but her friend had gone the other way and tagged her. When they were done they drank some juice.
7. Annie was in a park with her mother when a cute little bee landed on her hand. Annie could feel the tickle of the bee legs walking on her. Then the bee crawled onto a flower that Annie was holding. Annie held the flower tight and ran to her mom.
8. Roy was at the beach with his family. After a day of playing catch and running in the sand, Roy started walking back to the car. Buried in the sand was a piece of smoothed blue glass. Roy picked up the glass and put it in his pocket.
9. Cindy was making dinner for her family. She pulled the pot of spaghetti off the stove and walked over to the sink to drain it. The pot tilted and the spaghetti and water slid into the strainer. Cindy rushed over to the refrigerator to get the sauce.
10. Chad was collecting wood to build a tree house. Chad picked up a long plank of old wood that was perfect for the floor. The wood slid in his hand and a splinter came off. The splinter stuck into another piece of wood and broke off.
11. Julia was sitting on a folding chair in her new room working at her desk. Julia needed to shift closer to her desk so she reached down and pulled the chair forward. Just then a pile of books collapsed and ended up strewn across the floor.
12. Lauren slept on a new pillow last night that was firmer than she was used to. Lauren has had back problems ever since she had a bicycle accident. Lauren woke up in the morning with no back pain and she did not have to take any Advil.
13. Jay has a pet rabbit that he keeps in a cage. One day he fed the rabbit some carrots and then put the rabbit away. As he was walking past the cage his bare leg brushed by the fur of the rabbit that was sticking out. It felt soft on his thigh.
14. Bill was walking along a picket fence with his friend. Bill is in kindergarten and was trying to show his friend how fast he could walk. Bill walked to the end of the fence and jumped off. He then took off his shoes and left them hanging on the fence.
15. Cathy was on an Island in the South Pacific for her honeymoon. She walked down the beach and into the warm water. As Cathy waded out she stepped onto the soft sand, which squished through her toes. She sighed and wiggled her toes further in.
16. Steve and his friend were riding bicycles together. Half way down a hill Steve realized that his chain was broken. At the bottom of the hill Steve pulled over and got out his chain repair tool. He sat on the ground cradling the broken chain.
17. Suzie was riding in a cab to meet some friends for dinner. When she arrived at the restaurant Suzie opened the door and began to step out. Just then a child walking by gently bumped into Suzie and apologized quickly for brushing by her leg.
18. Eric was hiking in the mountains with his girlfriend. At a stream crossing Eric watched his girlfriend hop across the rocks and then began to follow. He stepped on the first rock and his boot came down hard on it, ripping off a swath of moss.
19. Angela was putting up posters in her new room. It is early Saturday morning and she was in her pajamas. As Angela stood back, she picked up a thumb tack that had fallen on the ground. She pushed the thumb tack through the poster into the wall.
20. Sonya was playing tag at school with her friends. While running around a corner, Sonya practiced sliding like she does in baseball. The knees of her jeans were now green with grass. Sonya brushed the grass off her knees and started laughing.
21. Larry was going to his first day of a new job. The job starts very early so Larry was extremely tired. Larry made himself some fresh juice and took a big drink. The juice was cool and Larry felt the tang of it on the soft tissue inside his mouth.
22. Brian was at home when a friend called to him from outside. Brian raised the window to talk to his friend. Suddenly the window curtain unrolled and Brian caught the soft fabric in his fingers. Brian rolled the curtain back up and tucked it in.
23. Lois was skiing for the first time. She was learning how to go down the easy slopes and was trying to keep her legs together. On her first run down a harder slope her legs stayed together and her knees stayed bent. Lois hooted and hollered.
24. Oscar was doing the dishes after dinner. He was talking with his friends while his hands were in the soapy water. Oscar reached his hand in and hit a soft sponge. He grabbed the sponge between his fingers and pulled it out of the dirty water.
25. Ron and his wife recently became a foster parents to a 10 year old girl. Ron promises her that he will be at a concert that she is playing in. On the way to the concert Ron buys some flowers. When he arrives the concert is just about to begin.
26. Aaron was going to visit his father. There was so much that he had never told his father, and he finally wanted to let him know how much he cared. When he got to his parents house delivery truck was there. His father was signing for a package.
27. Diane has a son with a balanced life. Her son is very comfortable with others but sometimes prefers to play on his computer. With her husband away at work, Diane is alone at home with her son. Diane got up and went to give her son a hug.
28. Shelley recently applied to Oxford; she has dreamed of going there all her life. One day a letter came from Oxford and Shelley tore it open. The letter confirmed that they received her application. Shelley shrugged.
29. Mark is an autistic child who was just given his first dog. He loves the dog very much and considers it his best friend. One day Mark’s father wakes him up and tells him that the dog needs a bath. Mark gets out of bed and ready for the bath.
30. John was on a hike with his girlfriend. He had an engagement ring in his pocket and at a beautiful overlook he proposed marriage. His girlfriend said that she would marry him and began crying. John held his new fiancee and looked at the ring.
31. Monica was visiting Anna, who had been her best friend for 60 years. When Monica arrived at her house, Anna’s son said that Anna just went out to get groceries and was not sure when she would be back. Monica sat to wait on the front porch.
32. Alex had wanted to ask Christy on a date for months. One day Mark walked up to her and asked her out. Christy said that she would be happy to go out with him. She accepted the flowers he had brought for her with a smile.
33. Rose was planning her upcoming wedding. She received a call from her fiancee. He told her that he was leaving town for business and would be back late. Rose hung up the phone and picked up the wedding catalogue she was reading.
34. Kevin took his son Zack to the doctor for a checkup. The doctor did a series of tests and came back to talk to the father and son. The doctor told them that Zack is perfectly healthy. He made an appointment to see Zack again in 6 months.
35. Katie was giving a piano recital and was waiting for her turn. All the children walked off the stage to their parents when they finished. Katies parents were sitting in the back of the room. When she finished her recital she walked and sat with her parents.
36. Lara just moved and is trying to make friends at a new school. While Lara was sitting in one of the bathroom stalls, a number of girls came in. The girls talked about how much they dislike the new teacher and then they left. Lara sat silently.
37. Lissa was excited to have a solo in her upcoming performance. Her father promised her that he would be there in the front row for the concert. When the concert comes her father was there to watch. After the show Lissa sat on the stage with her dad.
38. Chad plays on a soccer team. After one of their big games Chad asks his teammates if they want to go get some ice cream. All of his friends tell him that they are going out with their fathers. Chad and his father join all the others to get pizza.
39. Karen was pregnant and going in for her first check-up. The doctor ran a number of tests and came back in to talk with her. The doctor told Karen that her baby was healthy and developing normally. Karen thanked the doctor.
40. Lucas is a painter who just moved all of his beloved paintings to a new studio. One day on his way to the studio he hears music. Lucas drives up to find a parade out front of his studio; all of his work is being admired.
41. Mike is very much in love with his girlfriend. One day he goes to surprise her at work for lunch. Through the window of the building he sees his girlfriend walking with a computer tech. She stops and gives the tech her laptop to fix.
42. Alison lives by herself. Every week her son comes to visit her and they go out to lunch. Her son just got a new job and is enjoying life with his wife. One day Alison gets a call from her son who says that he will meet her out front.
43. Nina lives with her parents in a small house. Lately her parents have been watching TV at night. They try to keep it quiet, but Nina can hear it. One day Nina’s father tells her that she is old enough now and can stay up sometimes to watch TV.
44. Rudy had worked hard to get the job he has now. He can finally take care of his son and has almost saved enough to give him the gifts he wants for his birthday. Today he was called in to the office and his boss told him that he was doing a good job.
45. Judy lives with her teenage daughter. Her daughter wants to have new friends and invites a number of people to her 15th birthday party. Some new people show up and leave after a good night. Judy and her daughter go to their rooms to sleep.
46. Brett was taking his children to the family cabin. Brett was going to show his children all the family pictures and history. When they arrived the cabin was surrounded by snow. Last night it had snowed for the first time that season.
47. Cynthia lives with her husband and step-daughter, Julia. She loves Julia very much and often tries to help with her homework. One day Julia looked up from her work and asked Cynthia for some help. Cynthia helped and then sat down at the table.
48. Bobby was new to school and joined the volleyball team. Tonight is their first match. At the end of a close game the ball came right to Bobby for an easy play. He bumped it up and the game continued. His teammates ran around the court to play the ball.

Participants saw half of the stories involving low to high Pain/Suffering, and the complementary half of the matched stories involving no to low Pain/Suffering.

*Pain empathy localizer study:*

To define regions of interest involved in pain perception, we used data from a Pain Localizer study performed on an independent data set. In the Pain Localizer task, participants were told that they would be involved in a study investigating how the brain processes pain in the self and others, and were told that the study would involve two participants: one of the participants would experience painful electrical stimulation to the hand while in the scanner, and the other participant would experience painful electrical stimulation to the hand while being monitored with psychophysiological equipment. In fact, all participants were recruited for fMRI imaging, and the other participant was a confederate of the study.

The pain threshold of each participant was determined in the waiting room. A small electrode was attached to the back of the hand and a shock was delivered with a current generator. Voltage on the current generator was initially set to a level that was undetectable to the participant, and then was increased in small intervals until the participant reported feeling a sensation that was barely perceptible. This was recorded as the ‘non-painful’ shock level for the participant. Voltage was then increased in 5 V steps until the participant reported a shock that was ‘uncomfortable or mildly painful’. This was then recorded as the ‘painful’ shock level for the participant. The same procedure was repeated for the confederate. Confederate ‘painful’ shock levels were pre-determined to cause a visible twitch of the hand, and the number of steps between ‘non-painful’ and ‘painful’ shock levels for the confederate was matched to the number of steps between ‘non-painful’ and ‘painful’ stimuli for each participant. At the ‘painful’ shock level, confederates winced to express their discomfort with the stimulus.

During the experiment, the confederate stood at the back of the scanner. The confederate’s arm rested on a custom-made stand so his or her hand protruded into the visual field of the prone participant, between the back of the fMRI bore and the screen on which visual stimuli were presented. The screen was easily visible by both the participant and the confederate.

During the experiment, a picture of either the participant or the confederate (taken immediately before the study) appeared on the screen for 2 seconds to indicate who would be receiving a shock. A border then appeared around the picture for 2 seconds to indicate the level of shock: a light pink border indicated that the ‘non-painful’ shock would be delivered, and a dark red border indicated that the ‘painful’ shock would be delivered. Three 100 ms pulses were then delivered each second over a 4 s period. The target of the shock and shock level were pseudorandom. In all, participants and confederates each received 3 ‘painful’ shocks (SelfPain and OtherPain) and 3 ‘non-painful’ shocks (SelfNoPain and OtherNoPain) per run; the study lasted a total of 6 runs.

To define regions of interest, random effects analyses were performed on the Pain Localizer Experiment, using a threshold of p < 0.001 (voxel-wise, uncorrected), and a cluster threshold of k>10 on the data from 13 participants. ROIs were defined as the group of superthreshold voxels within a 9 mm sphere of the peak coordinates in each region.
